# Preparation and Characterization of Immediate-Release Tablets Containing Calcium Lactate Recovered from Chemically Recycled Poly(lactic acid) 3D Printing Filaments

**DOI:** 10.3390/polym18192422

**Published:** 2026-10-04

**Authors:** Olivia A. Attallah, Dario Brandao, Jacqueline Tran, Irvin Rivera, Cindy A. Thomas-Charles, Michael R. O’Krepky, Mathew Mannon

**Affiliations:** 1 Chemistry Department, University of Hartford, West Hartford, CT 06117, USA; 2 Geoscience Department, Union College, Schenectady, NY 12308, USA; 3 Biology Department, University of Hartford, West Hartford, CT 06117, USA; 4 Chemistry Department, Union College, Schenectady, NY 12308, USA

**Keywords:** poly lactic acid, depolymerization, Ca lactate, immediate release tablets

## Abstract

Polylactic acid (PLA) is one type of biodegradable plastic that can replace single-use plastic. Nevertheless, it still poses the problem of accumulation in the environment due to the slow biodegradation rate. Developing simple yet effective methods for PLA recycling and upcycling can pave the way for a circular economy and help partially solve the plastic pollution problem. In the present work, PLA filaments of different colors were chemically recycled in alkaline solutions of C_1_-C_3_ primary alcohols under mild experimental conditions (75 °C for 30 min, 1 atm). The effect of various process parameters such as depolymerization solvent media and PLA pigmentation on the percentage of PLA depolymerization and Ca lactate monomer yield was studied. PLA filament size was also investigated as a factor affecting the kinetics of PLA depolymerization. As a monomer, Ca lactate was evaluated by different characterization techniques including Fourier transform infrared spectroscopy, Nuclear Magnetic Resonance, and Atomic Absorption Spectroscopy. In addition, the powder quality was tested against US Pharmacopeia standards for acidity, heavy metal presence, hydration state, and calcium content. The recycled Ca lactate was formulated with other ingredients into Ca lactate tablets via direct compression to serve as a calcium source for preventing and treating calcium deficiencies. The tablet characteristics were compared against commercially available Ca supplements with respect to shape consistency, uniformity of calcium loading, immediate release, and cytotoxicity. The obtained findings suggest that there exists a viable, environmentally friendly avenue for utilizing PLA recycling products in the development of compounds with potential applications in the pharmaceutical domain.

## 1. Introduction

As of 2025, the annual global production of plastic reached an all-time high at 450 million metric tons produced and is projected to climb with each subsequent year [1]. A large amount of this plastic is or will be discarded into landfills, where it often makes its way into ecosystems and environments, acting as a harmful pollutant. The main concerns with these plastics are the additives in them, which can take hundreds if not thousands of years to decompose [2]. As such it is important that future studies focus on discovering new and innovative ways of reusing and recycling current plastic materials and waste. The development of biodegradable plastic polymers such as poly(lactic acid) (PLA) that are derived from bio-renewable sources seeks to combat this issue. Nevertheless, despite PLA’s efforts to greenify the components and production of everyday plastics, the material itself can still contribute to the aforementioned harm if not properly disposed and recycled. With growing concerns surrounding the fate of plastics like PLA, it is imperative that further research is conducted to establish faster and more environmentally friendly methods of recycling and upcycling these materials.

Chemical recycling focuses on the deconstruction of the chemical structure of materials such as plastics and the subsequent transformation of the material into a potentially safer, less toxic, biodegradable, or economically profitable product. Our previous work concerning the alkaline hydrolytic depolymerization of Polymaker PLA fibers in 50% ethanol revealed a cost-effective and energy-efficient route towards producing a good-quality monomer as a product [3]. The lactic acid produced from the depolymerization process was reacted with excess calcium carbonate to form Ca lactate salt that was eventually precipitated out in ethanol and separated. Both the PLA depolymerization and Ca lactate extraction were optimized via Box–Behnken Design and the findings of these experiments were the basis for the current study. Bearing in mind that the chemical recycling of PLA plastics is quite varied and rests upon several key factors. Our current study aimed to examine the effect of varying key variables such as type of depolymerization solvent, plastic color, and filament size on the percent degradation of PLA, and the yield and quality of the calcium lactate product.

First, the type of solvent used is evidently integral to the degradation process. Investigating the effect of varying the organic chain length of the primary aliphatic alcohol used as medium for the depolymerization reaction was of particular interest. For instance, Sanda et al. [4] studied the alkaline hydrolysis of polyethylene terephthalate (PET) in alcohols of different chain lengths such as methanol, ethanol and 1-propanol. The authors found that increasing the aliphatic chain length decreased the degradation of PET; under optimum conditions, the conversion % of PET was calculated to be 92.2%, 91.7% and 44.50% for methanol, ethanol and 1-propanol, respectively.

Particle size is also a significant factor affecting the degradation rate of any chemical reaction. Generally speaking, reducing the particle size of a material exposes more of its surface area, which increases its potential for reacting with reagents [5]. Given that efficiency with time and energy are top priorities in chemical recycling processes, it makes sense that one considers adjusting the particle size in order to save these precious resources. Thus, studying the effect of filament size on the kinetics of degradation of PLA was another focus of our study.

Pigments, dyes, and other additives are commonly added to plastic products to meet consumer aesthetic needs. However, the presence of these additives and pigments in plastic products oftentimes affects the rate of mechanical degradation of these products and exacerbates their overall impact on ecosystems and the organisms that inhabit them. It is quite difficult to discover a particular solvent or technique that is effective at removing the various mixtures of pigments and unwanted additives [6]. The complex structures of these chemical additives are likely to blame. In addition to being detrimental to the environment, these additives and pigments also pose a potential problem to the chemical recycling process—the synthesis of a good quality product.

The stained products that are made from plastics containing pigments are typically less desirable by manufacturers and are thus considered to be less valuable. From a financial point of view, these stained recycled products also sell for much less than their non-stained counterparts, making the hassle of removing added pigments much more than a cosmetic issue [6]. In the present study, we were particularly concerned with the color of the calcium lactate produced from the depolymerization of colored PLA filaments. Our thought was that the added pigments might make their way into the calcium lactate end-product, thus lowering its quality. Evaluating the effect of PLA pigmentation is accordingly a crucial step to confirm the good quality of the produced Ca lactate and its suitability for upcycled applications.

Calcium lactate (C_6_H_10_CaO_6_) is an FDA-approved non-toxic organic salt with a variety of industrial and commercial applications. The white crystalline solid, which is formed from two lactate anions paired to one calcium cation is readily soluble in water, making it a very versatile product. Calcium lactate is produced via the neutralization of lactic acid with calcium carbonate [7] and most commonly exists in its pentahydrate form [8]. In industry, the lactic acid component forming Ca lactate is typically produced through the bacterial fermentation of simple carbohydrates and is commonly utilized in the production of the poly(lactic acid) (PLA) bioplastic [9]. Global lactic acid production is estimated to rise to 2.66 million metric tons [10], with an estimated associated market value of 2.8 billion US dollars in 2029 [11]. Such figures suggest general interest in lactic acid in both chemical recycling and bioplastic production fields. Commercially, the salt of Ca lactate is used as a thickening and flavoring agent and serves as a frequent source of calcium in many daily supplements [8]. Some studies also provide evidence for calcium lactate’s role as an anti-carcinogen and anti-microbial [12].

Recent research on the potential pharmaceutical and agricultural applications of calcium lactate corroborates this claim. For instance, in a study conducted by Mititelu et al., calcium lactate was synthesized from the extracted calcium of mussel shells and subsequently used as a primary ingredient in rapid-release Ca lactate tablets [12]. Opting to use the same neutralization method of synthesis via reacting lactic acid with Ca from mussel shells, Mititelu et al. were able to produce a high-quality, inexpensive, easily synthesized calcium lactate tablet that complies with compendial standards. Their findings prove that there exists a viable, environmentally friendly avenue for utilizing and extracting calcium lactate for use in the pharmaceutical industry.

Agricultural uses of calcium lactate also appear to show real promise. Khani et al. examined the effect of applying dissolved calcium lactate to lettuce plants (*Lactuca sativa* L.) that were experiencing water stress [13]. When treated with a solution of 1.5 g L^−1^ calcium lactate, the lettuce plants produced higher crop yields [13]. In addition, the calcium lactate-treated plants had increased leaf water status and antioxidant capacities compared to the untreated samples.

Considering calcium lactate’s multifaceted roles as a food additive, pharmaceutical agent, and potential fertilizer is profitable and worth exploring. The present study will not only investigate the additional factors that can affect the chemical recycling of PLA but will also focus on the upcycling of the Ca lactate obtained from the PLA depolymerization. The recycled Ca lactate will be evaluated against US pharmacopeial standards to be used in the manufacture of Ca supplements in the form of tablets. The tablets will then be tested for their release kinetics and potential toxicity against THLE-2 human hepatocytes to serve as a potential Ca supplement.

## 2. Experimental 

### 2.1. Chemicals and Reagents

Commercialized FAHKNS polylactic acid (PLA) 3D printer filaments were obtained from FAHKNS store (US-based Amazon warehouse, USA). The filaments are 1.75 ± 0.02 mm in diameter, have an average molecular weight (Mn) of 183,000 g/mol and come in different colors such as black, white, yellow, red and blue. The filaments were cut into different lengths according to the experiment’s requirements. Absolute ethanol (100%), sodium hydroxide (NaOH) pellets, hydrochloric acid (HCl) (34%), and calcium carbonate (CaCO_3_) were purchased from Sigma-Aldrich (NY, USA). Food-grade Ca lactate was obtained from Modernist Pantry store through Amazon (NY, USA). Methanol (100%), isopropyl alcohol (100%), magnesium stearate, and sodium starch glycolate were supplied by Fisher scientific (USA). Avicel^®^ PH302 was provided as a gift from ROQUETTE—Wilmington (DE, USA).

### 2.2. Alkaline Hydrolysis of PLA in Alcoholic Media

The hydrolysis of PLA was performed according to our previously published work with slight modification [3]. The purpose of these experiments was to extend the procedure optimization process we initiated in our previous work [3] by evaluating the effect of pigmentation and primary aliphatic alcohol chain length on the efficiency of PLA depolymerization and yield of lactate salt.

The reaction was carried out in a 250 mL 2-necked flat-bottom flask where 2.5000 g of NaOH was dissolved in 20 mL of 50% primary (C_1_-C_3_) aliphatic alcohol. About 5.0000 g of PLA was added to the mixture and the reaction was refluxed under magnetic stirring at 75 °C for 30 min. After the reaction was complete, the flask was cooled to room temperature and the unreacted PLA was collected by filtration and dried at 70 °C overnight. The plastic conversion (%) was calculated using Equation (1):

Plastic conversion (%) = (W_1_ − W_2_)/W_1_ × 100 (1)

where W_1_ was the initial weight of PLA sample and W_2_ was the weight of the unreacted residue obtained post PLA depolymerization reaction.

The pH of the cooled filtrate was adjusted using concentrated HCl to 3.33 to convert the sodium lactate salt to lactic acid. The solution was completed to 80 mL with deionized water and powdered CaCO_3_ was then added portion-wise to the solution under vigorous stirring to extract lactic acid in the form of Ca lactate until the pH was neutralized and excess CaCO_3_ precipitated out. The solution was then left under stirring and gentle heating at 60 °C for 15 min and the excess CaCO_3_ was filtered out. The obtained filtrate was concentrated to 25 mL and the Ca lactate was precipitated as white powder by pouring the filtrate into absolute ethanol and leaving overnight under gentle stirring. The obtained precipitate was separated, washed and dried in the oven at 40 °C. The resultant Ca lactate yield was calculated as mmol of Ca lactate (Equation (2)).

(2) $$\mathrm{Yield}\;\mathrm{of}\;\mathrm{Ca}\;\mathrm{Lactate}\;(\mathrm{mmol})=\frac{\mathrm{weight}\;\mathrm{of}\;\mathrm{obtained}\;\mathrm{Ca}\;\mathrm{lactate}\;(g)}{\mathrm{Molar}\;\mathrm{mass}\;\mathrm{of}\;\mathrm{Ca}\;\mathrm{lactate}\;(218.22\;g/\mathrm{mol})}\times 1000$$

### 2.3. Characterization of Obtained Ca Lactate According to USP Protocols for Ca Lactate Powder

The USP protocol has stated specific criteria for Ca lactate powder that can be accepted for drug applications. The assays were followed as described in the USP monograph for Ca lactate [14] with slight modifications to ensure more accurate and precise results.

The chemical structure and purity of the obtained Ca lactate were confirmed by recording its Fourier transform Infrared (FTIR) and ^1^H-NMR spectra and comparing them against those of commercialized standard of Ca lactate.

Acidity of the Ca lactate powder was tested by titrating a 10 mL solution of Ca lactate sample (500 mg) against 0.05 M NaOH with phenol phthalein as indicator. According to USP, the white powder should contain no more than 0.45% lactic acid.

The presence of heavy metal impurities was analyzed by ICP/MS by dissolving 0.5 g of Ca lactate in 2.5 mL of 1 N acetic acid, and dilute with water to 25 mL. The solution was diluted and analyzed according to a previously established protocol. According to USP, the white powder should contain no more than 20 ppm heavy metals in total.

To determine the hydrate form of Ca lactate, 500 mg of Ca lactate sample was distributed evenly in a suitable weighing dish to a depth of no more than 3 mm and dried at 120 °C for 4 h. The weight loss % is recorded and compared against reported weight loss % that corresponds to specific hydrate forms of Ca lactate [14].

Detailed instrumental procedures are provided as Supplementary Material in (S1 Instrumentation).

### 2.4. Quantitation of Ca Lactate via Atomic Absorption Spectroscopy (AAS)

To quantify the amount of Ca lactate in the obtained white powder, an in-house Atomic Absorption Spectroscopy (AAS) assay was developed and validated to replace the complexometric titration assay proposed by the USP. The developed AAS not only provides faster, more accurate analysis of Ca^2+^ content (equivalent to Ca lactate) but is also user- and environmentally friendly because little waste is generated from the analytical procedure.

A Perkin Elmer PinAAcle 500, AAS (CT, USA) operated with Syngistix software version 4.0 (USA) was used. To start the flame, acetylene (2.5 L/min) and air (10 L/min) were selected as the fuel and oxidant, respectively. A calcium analysis method was set up in the machine; the calcium lamp was selected to operate at 422.7 nm wavelength. Maximal absorbance was achieved by adjusting the position of the burner’s head and the machine was autozeroed with DI water.

#### 2.4.1. External Calibration Method Development and Validation for Quantitation of Ca^2+^

A 400 ppm stock solution of Ca^2+^ was prepared by dissolving CaCO_3_ in slightly acidified water. A 100 ppm stock solution of Ca^2+^ was then prepared from that solution. Six working standard solutions over the range 4–14 ppm Ca^2+^ were prepared in 100 mL volumetric flasks. The absorbance of the six standards was recorded and used to construct a linear calibration curve for Ca^2+^. The limit of detection (LOD) and limit of quantification (LOQ) were estimated according to ICH guidelines Q2 R1.

The recovery and accuracy study involved the analysis of five different Ca^2+^ standards of concentrations ranging between 5 and 13 ppm. The accuracy was expressed as a percentage recovery of Ca^2+^ and calculated using Equation (3). The accuracy should be within 98–102%.

(3) $$\%\;\mathrm{Recovery}\;\mathrm{of}\;{\mathrm{Ca}}^{2+}=\left(\frac{\mathrm{Calculated Ca concentration}-\mathrm{theoretical Ca concentration}}{\mathrm{theoretical Ca concentration}}\right)\times 100$$

The recovery and precision study involved the analysis of three samples of 4.0, 8.0 and 12 ppm Ca^2+^ standards. These samples were analyzed, and the process was repeated the following two day for the interday precision study. The results were expressed as a percentage of recovery.

The intraday precision was assessed by averaging the recoveries obtained for each concentration for one day and calculating its standard deviation. The intraday precision was assessed by averaging the recoveries obtained for each concentration for the three days and calculating their standard deviation. In both studies, the standard deviation of recoveries should not exceed 2%.

#### 2.4.2. Determination of Ca Lactate Content in the Recycled White Powder

For sample analysis, 100 mg of Ca lactate white powder was dissolved in 100 mL DI water and was diluted by a factor of 20. The solution was analyzed via AAS and the concentration of Ca^2+^ in ppm was determined using the developed external calibration method. The percentage of Ca lactate in the white powder was determined using Equation (4). According to USP, the white powder should contain 98 ≤ x ≤ 101% Ca lactate [14].

(4) Ca lactate in white powder (%)=((calculated concentration of Ca ± absolute uncertainty in ppm)×20((weighed Ca lactate powder mg×atomic mass of Cagmolmolar mass of Ca lactate gmol)/0.1  L))×100

### 2.5. Ca Lactate Tablets Formulation

#### 2.5.1. Tablets Formulation Studies

For developing an optimum pharmaceutical form while considering that the active ingredient dosage should constitute a big portion of the tablet, the excipients must be carefully selected to perform efficiently in low quantities, as it is not compliant to manufacture high-weight tablets. Based on previous studies of Ca lactate synthesized from mussel products [12], microcrystalline cellulose 302 as a filler excipient, sodium starch glycolate as a super disintegrant and magnesium stearate as a lubricant were selected as formulation excipients for our recycled Ca lactate.

The active ingredients (standard calcium lactate and recycled calcium lactate from black PLA filaments) were each mixed with the direct compression excipients with different ratios as illustrated in Table 1 with the aim of maintaining good tablet mechanical properties and maximizing the active ingredient content. The raw materials were accurately weighed and were mixed and homogenized in a blender for 5 min at 15 rpm, at room temperature. Over the blend, the magnesium stearate was added and lubricated for 1 min.

The immediate-release tablets were manufactured by direct compression in a TDP 0—V2 desk top tablet press (LFA Machines—TX, USA), equipped with 7 mm flat punches, adjusted to a 6 mm tablet thickness with an applied compression force of 3.1 kN.

#### 2.5.2. Characterization of the Formulated Tablets

After manufacturing, the obtained tablets were tested to determine the quality characteristics of the final products, as a preliminary trial to prove the applicability of the recycled Ca lactate in the pharmaceutical field and to choose the best formulation ratio that will provide acceptable tablet size, shape and mass uniformity and the highest calcium content.

##### Tablet Size and Mass Uniformity

The uniformity of the tablets’ diameter and thickness was determined by measuring the dimensions of 5 tablets of each formulation. The weight variation was evaluated by individually weighing 5 tablets of each formulation, then calculating the average mass. The tablets comply with the test if no more than 1 individual mass is outside the limits of 85–115% of the average mass [15]. Tablet hardness, mechanical strength and friability were not tested in the current study due to the limitations of our lab instrumentation. Such tests will be performed in future studies. Nevertheless, the tablets showed good consistency, uniform shape and were not crushable or powdery when held in the hand.

##### Tablet Calcium Content

The calcium content of tablets was determined using an AAS-developed assay. The test was performed on 3 tablets of each formulation. Each tablet was weighed, crushed and grinded with a pestle in a mortar. Then, 100 mg of the obtained tablet powder was dissolved in 50 mL water by stirring for 20 min. The undissolved residue was filtered out and the filtrate was poured in a 100 mL volumetric flask and DI water was added until a quantity of 100 mL was reached. The concentration of Ca^2+^ ions was determined using the external calibration method on AAS and reported as the average concentration in mg of Ca^2+^ per tablet.

### 2.6. In Vitro Dissolution Kinetics of Recycled Ca Lactate Tablets

The Ca^2+^ release kinetics were studied using USP basket Apparatus I (MSE Pro 3pcs disintegration basket (MSE supplies-AZ, USA)) on the optimized formulation of Ca lactate tablets synthesized from the recycled Ca lactate. The apparatus temperature was adjusted at 37 ± 0.5 °C, with a rotating speed of 100 rpm, and the test was performed on 3 tablets of each formulation. The amount of dissolved calcium lactate in 500 mL of DI water was determined after 0, 5, 10, 15, 20, 25, 30, 35, 45, 60, 90 and 120 min, by the AAS assay. After each specified time, a 5 mL sample was collected, filtered and analyzed in the AAS [16]. The release kinetics was studied as the percentage of Ca^2+^ released per time in minutes and fitted against different kinetics model equations to determine the best-fitting model for the release of calcium from the formulated tablets.

### 2.7. Cytotoxicity Evaluation of Recycled Ca Lactate Tablets

*Preparation of tablet extract*: Calcium lactate tablet extract was prepared by dissolving one tablet (26–29 mg calcium lactate) in 10 mL sterile Milli-Q water with continuous stirring for 30 min. The resulting extract was sterilized by passing it through a 0.22 µm syringe filter (Fisherbrand, USA) and then diluted in culture medium to the indicated treatment concentrations.

*Cell line and culture*: THLE-2 liver epithelial cells (ATCC, USA) were maintained in Bronchial Epithelial Cell Growth Medium (BEGM; Lonza, USA) supplemented according to the manufacturer’s recommendations. Cells were maintained at 37 °C in a humidified atmosphere containing 5% CO_2_.

*Lactate dehydrogenase (LDH) activity assay*: THLE-2 cells were seeded in 24-well plates at 2.0 × 10^5^ cells/well and cultured to approximately 80% confluency before treatment. Cells were exposed for 48 h to supplemented BEGM containing calcium lactate tablet extract at nominal calcium lactate concentrations of 0.0165, 0.033, or 0.066 mg/mL, designated as the low-, mid-, and high-dose groups, respectively. Concentrations were selected to bracket a nominal tablet-equivalent exposure derived from the calcium lactate content (26–29 mg/tablet) and recommended daily intake of six tablets. The selected concentrations were used to establish a defined in vitro tablet-equivalent exposure range and were not intended to represent pharmacokinetically equivalent circulating concentrations following oral administration.

Cytotoxicity was quantified by measuring LDH release into the culture supernatant using the Invitrogen CyQUANT LDH Cytotoxicity Assay Kit (Invitrogen, USA) according to the manufacturer’s protocol. Spontaneous and maximum LDH-release controls were included, and percent cytotoxicity was calculated relative to these controls. Absorbance was measured at 490 nm with a reference wavelength of 680 nm using an Infinite M Nano+ microplate reader (Tecan, NC, USA).

## 3. Results and Discussion

### 3.1. PLA Solvolytic Depolymerization Study

**Figure 1:**
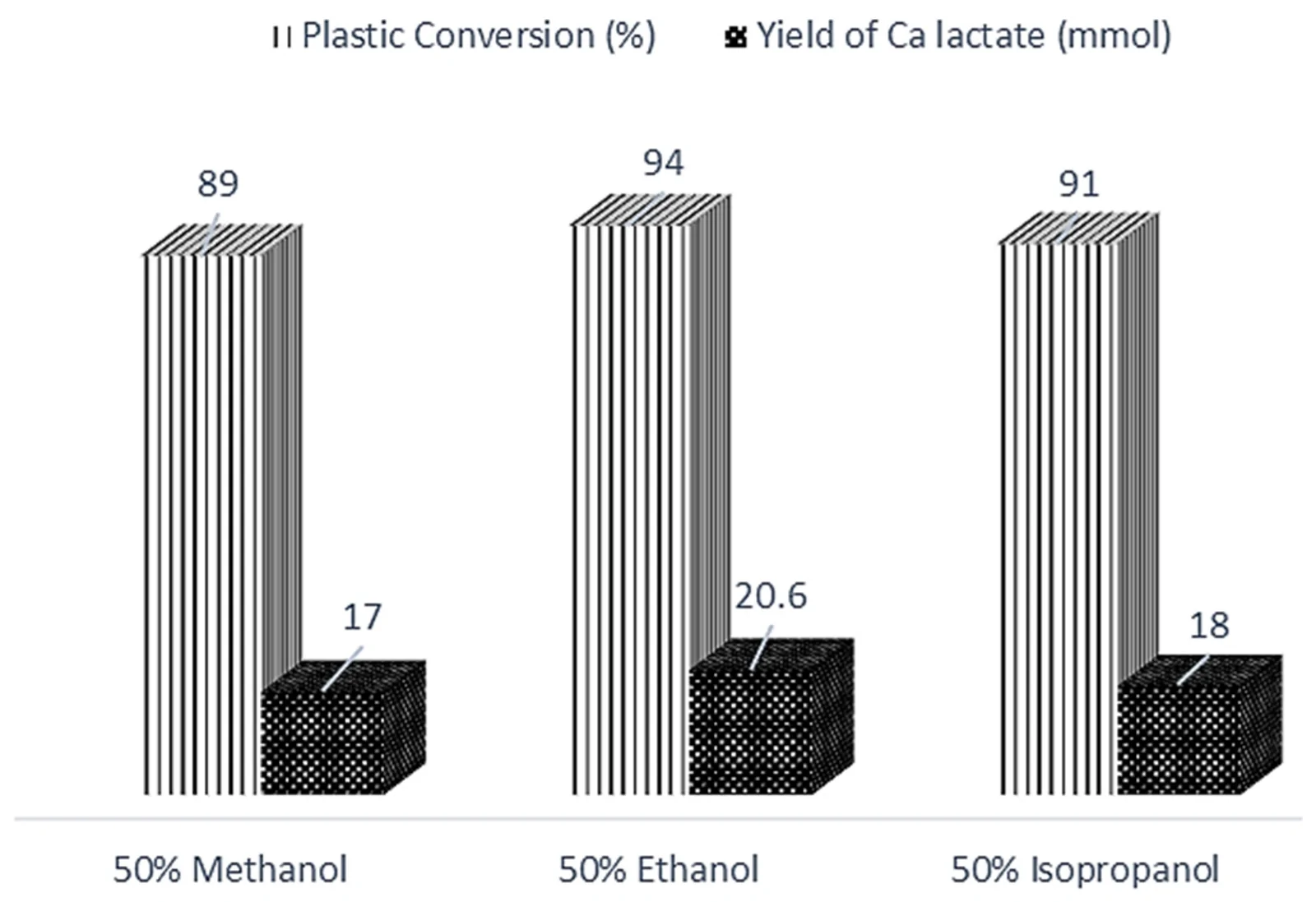
Effect of primary aliphatic alcohol chain length on PLA conversion and yield of Ca lactate (reaction conditions: 5 g PLA, 2.5 g NaOH in 20 mL 50% alcohol, time: 30 min, temp. 75 °C).

**Figure 2:**
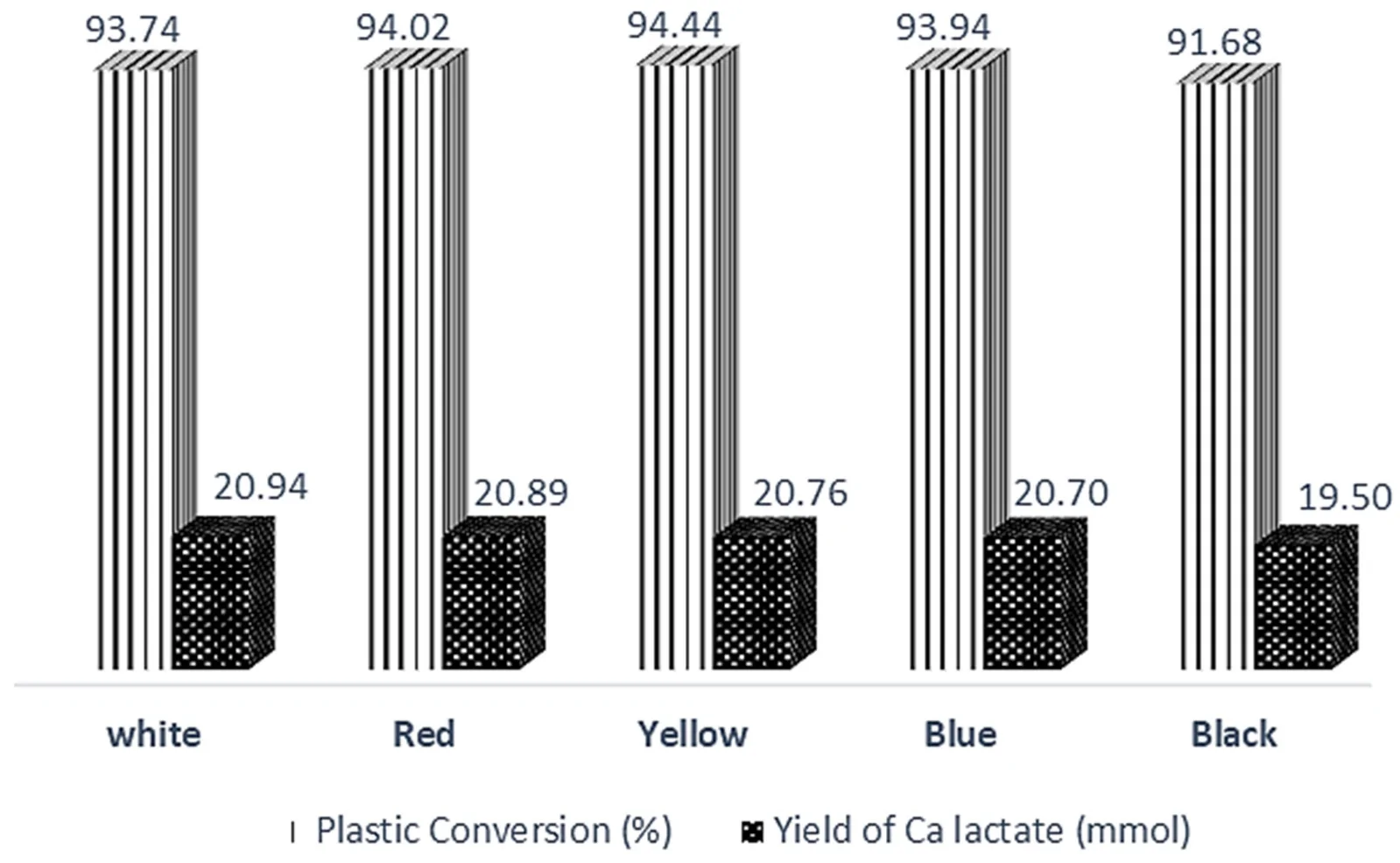
Effect of PLA filament pigmentation on PLA conversion (%) and yield of Ca lactate. (reaction conditions: 5 g PLA, 2.5 g NaOH in 20 mL 50% ethanol, time: 30 min, temp. 75 °C).

The results of the depolymerization of PLA filaments in 50% alcohols are presented in Figure 1 and Figure 2. The extent of decomposition of PLA in 50% ethanol was found to be greater than what was observed for isopropanol and methanol media. As shown in Figure 1, an average of 94 ± 1% degradation of PLA was obtained in ethanol, while 91 ± 2% and 89 ± 3% were observed for isopropanol and methanol, respectively.

From the theory of PLA alkaline solvolysis, the expected primary product is lactic acid which in our study was further treated with CaCO_3_ to produce Ca lactate. For each experimental run, a white powder was obtained and its yield was quantified. The yield of Ca lactate for ethanol showed a slightly higher average value (20.6 mmol) than that of isopropanol (18 mmol) and methanol (17 mmol) which confirms the superiority of ethanol as a type of aliphatic alcohol in determining the extent of PLA depolymerization and the yield of Ca lactate.

Such results raise a question of why ethanol in particular performs slightly better than isopropanol (bulkier hydrophobic chain) followed by methanol (shorter chain length). Although computational modeling for the alcohol–polymer interaction would have provided better insights (and we plan to do this in future work), as stated in our previous work on PLA degradation, the mechanism of action of the alcohol lies in its ability to interact with the polymer chain via intermolecular forces (London dispersion forces and hydrogen bonds) [3]. This interaction causes the swelling and/or partial dissolution of the PLA, allowing the polymer to be exposed for the diffusion of NaOH and water into the polymer matrix to break down its chain. Theoretically, ethanol offers greater intermolecular forces (specifically London dispersion forces) interaction with the hydrophobic polymer chain of PLA than the bulky isopropanol and the short-chain methanol and accordingly results in slightly higher PLA depolymerization and Ca lactate yields than the other aliphatic alcohols.

The effect of PLA filament pigmentation on the extent of PLA depolymerization and yield of Ca lactate was also studied. Figure 2 indicates that the difference in PLA filament pigmentation did not have a significant effect on PLA depolymerization and yield of Ca lactate. For instance, the PLA depolymerization in 50% ethanol ranged between 91 and 94 for all the treated PLA filament pigments, with a Ca lactate yield of 19.5–21.0 mmol. The same trend was observed for the PLA depolymerization percentage and yield of Ca lactate in methanol and isopropanol. Such results indicate that the presence of the alcohol in the depolymerization medium could solvate the pigments and prevent their interaction with sodium hydroxide or water during their attack on the PLA polymer chain.

### 3.2. Kinetics of Depolymerization of PLA Filaments

In this study, the kinetics of the solvolytic depolymerization of PLA in 50% ethanol under conventional heating were studied to calculate the hydrolysis rate constants at different PLA filament sizes (0.5, 1.0 and 2.0 cm length) at constant temperature. In our previous work, the kinetics of constant PLA filament size (2.0 cm) degradation were studied at different reaction temperatures and first-order reaction kinetics were adapted for the PLA depolymerization. In the current study, first-order reaction kinetics were also assumed to control the depolymerization of PLA filaments with respect to PLA amount in 50% ethanol, and reaction rate constants were calculated using first-order kinetics equations (provided as Supporting Information S2).

**Figure 3:**
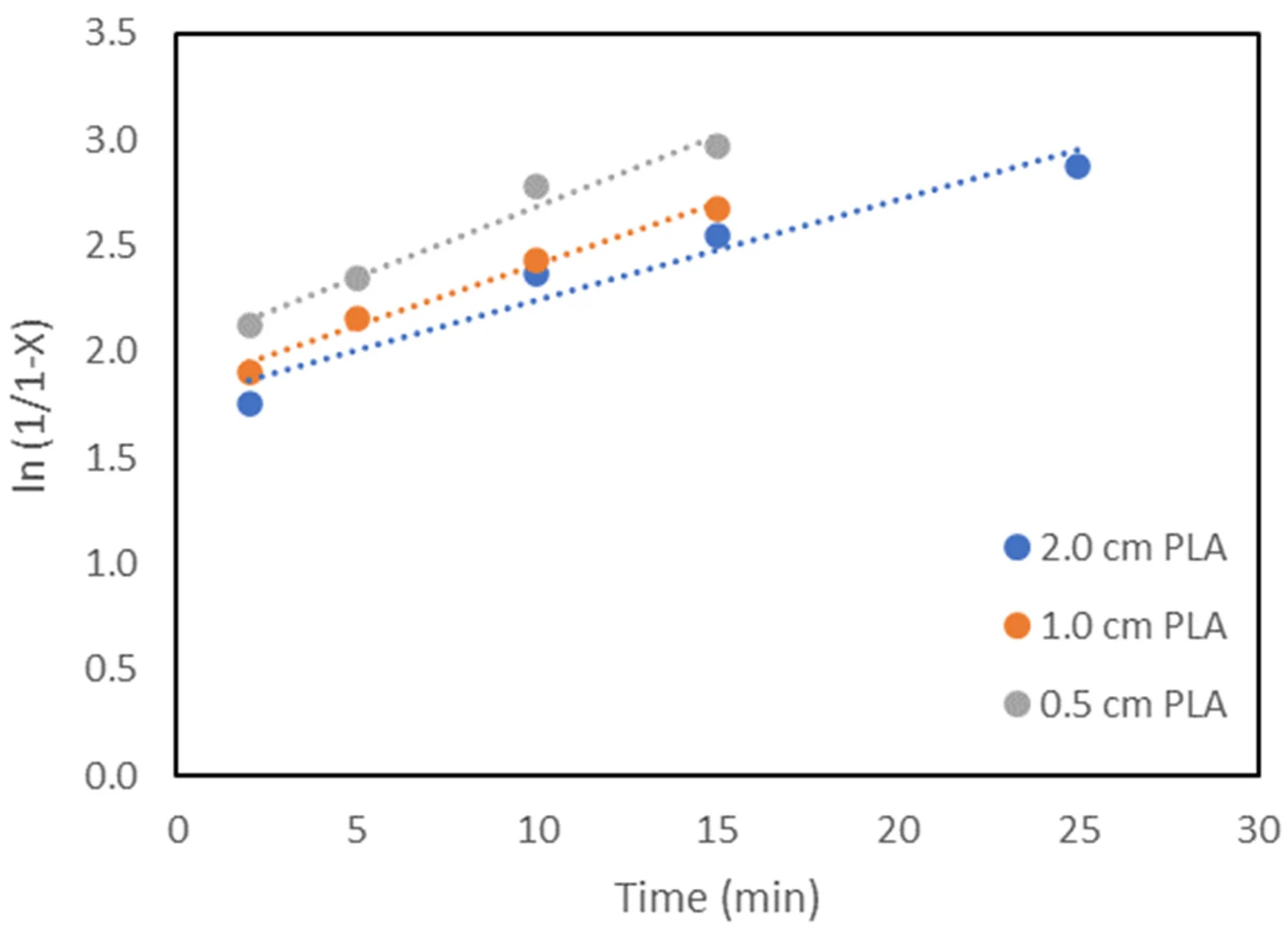
Effect of the filament size on the rate of PLA conversion (reaction conditions: 5 g white PLA, 2.5 g NaOH in 20 mL 50% ethanol, temp. 75 °C).

The influence of filament size on the reaction rate of PLA depolymerization in 50% ethanol is represented in Figure 3, and the values of rate constants (k) corresponding to each size are listed in Table 2. The increase in filament size from 0.5 cm to 2.0 cm resulted in a decrease in the rate constant from 0.0671 to 0.0474 min^−1^. This confirms the hypothesis that smaller particle size increases the rate of the reaction due to increased surface area of interaction with the depolymerizing agents, which eases their attack on the polymer chain and breaks it down. Correlation coefficient (R^2^) values higher than 0.94 were obtained from the linear regression of the straight lines at the different filament sizes, indicating that first-order reaction kinetics were followed where the PLA concentration dictates the rate of its solvolytic depolymerization in 50% ethanol.

**Figure 4:**
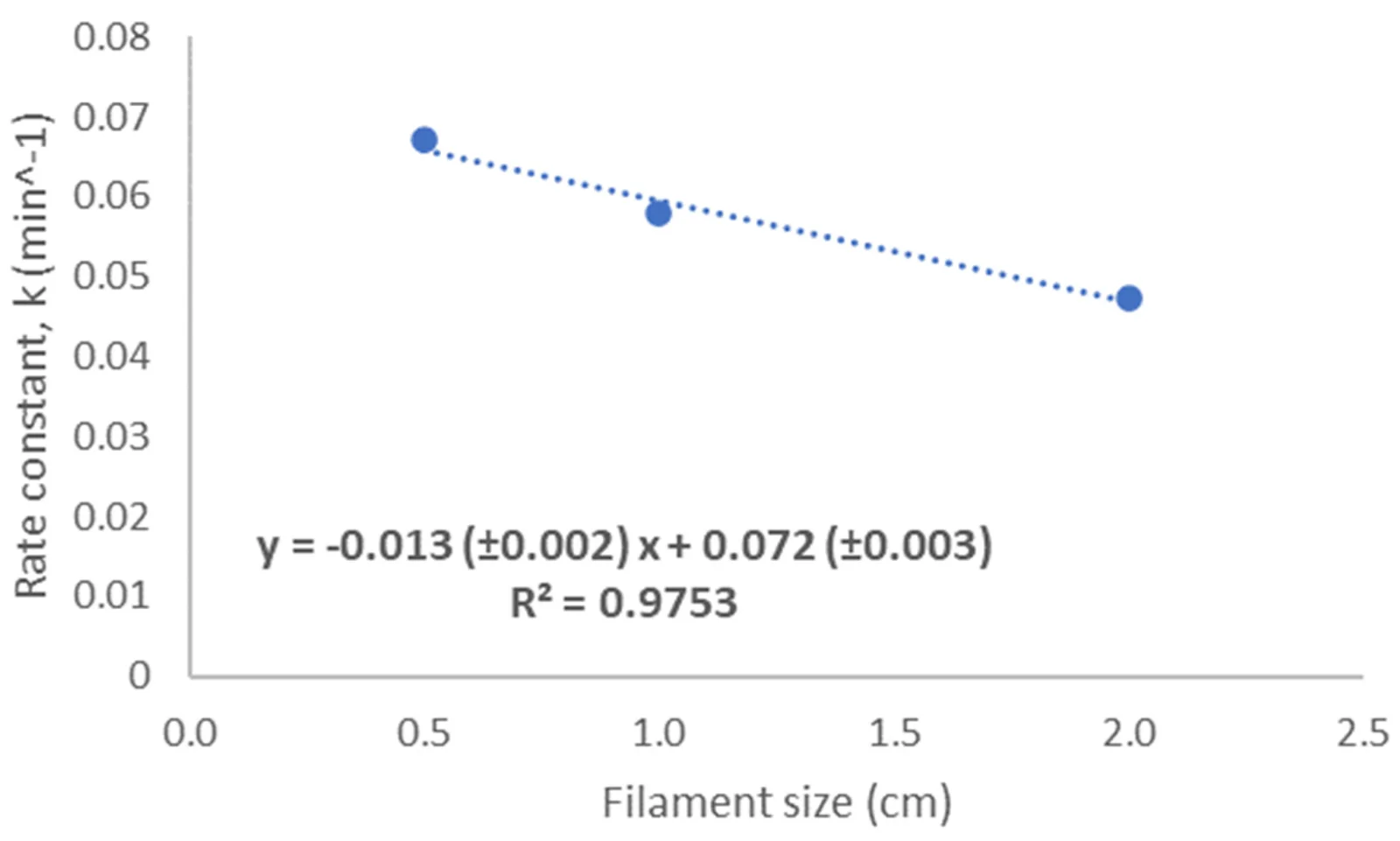
Linear regression plot for the rate constants (k) of PLA depolymerization against PLA filament size in cm.

Another thing to be considered while evaluating the effect of filament size on the solvolytic depolymerization of PLA is the linearity of the rate constant (k) values upon varying the filament size. As shown in Figure 4, there was an inverse linear relationship between the filament size and the rate constant for PLA depolymerization with an R^2^ value of 0.9753. Such findings could help with the prediction of the effect of filament size on PLA depolymerization rates using the obtained linear regression Equation (5) from the current study,

Rate constant (k) = [(−0.013 ± 0.002) × PLA filament size (cm)] + (0.072 ± 0.003)(5)

### 3.3. Characterization of Ca Lactate According to USP

The recycled Ca lactate obtained from the depolymerization of PLA filaments of different pigmentation in primary aliphatic alcohol media was characterized using various analytical techniques to ensure that its quality matches that required by standard USP protocols, except for the limits of magnesium and alkali salts and volatile fatty acids [14].

**Figure 5:**
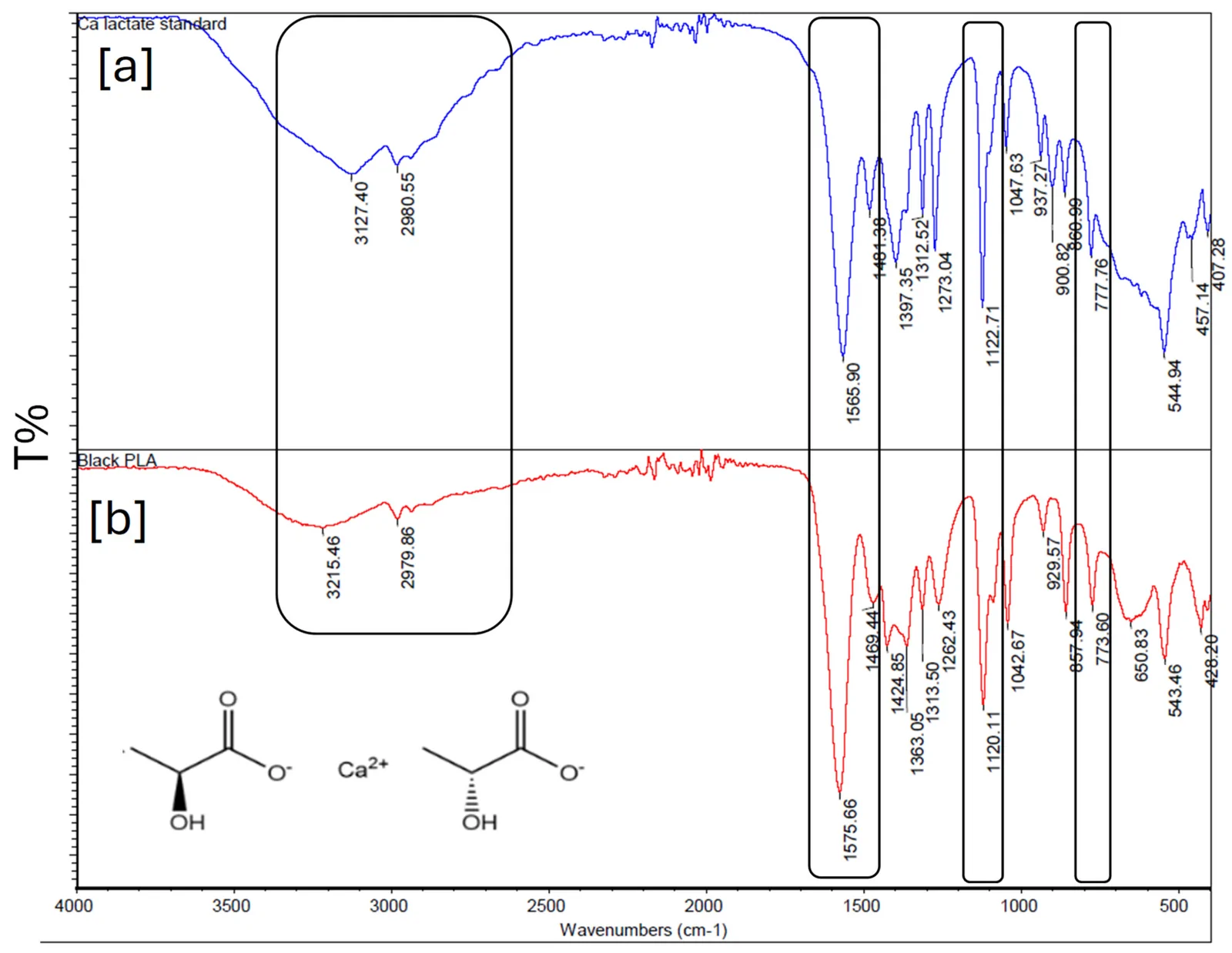
FTIR spectra of (**a**) Ca lactate standard and (**b**) recycled Ca lactate obtained after depolymerization of black PLA filaments in 50% ethanol.

For confirmation of powder identity, FTIR analysis was performed and the recorded IR spectral peaks matched that of standard Ca lactate as elaborated in Figure 5. Characteristic IR peaks for Ca lactate are observed as a wide peak ranging from 3126 to 2978 cm^−1^ denoting the carboxylic acid OH group; the strong and narrow peaks at 1569 cm^−1^ and 1481 cm^−1^ are characteristic for the asymmetric and symmetric stretching vibrations of the carboxylate (COO^−^) group, respectively; while the C–O bond present in the carboxylate salt appears as the peak at 1273 cm^−1^ [3]. The presence of these peaks confirms that the lactate is in its dissociated, salt form (bound to calcium) rather than its free, undissociated carboxylic acid form (which would show a peak around 1700–1750 cm^−1^).

All recycled Ca lactate samples obtained from different PLA pigmentation or using different aliphatic primary alcohols as solvolysis media showed similar peaks. The presence of the same functional groups in all the samples indicates that they are typically related to the Ca lactate molecular structure (the FTIR spectra are provided as Supplementary Material Figures S1–S3).

**Figure 6:**
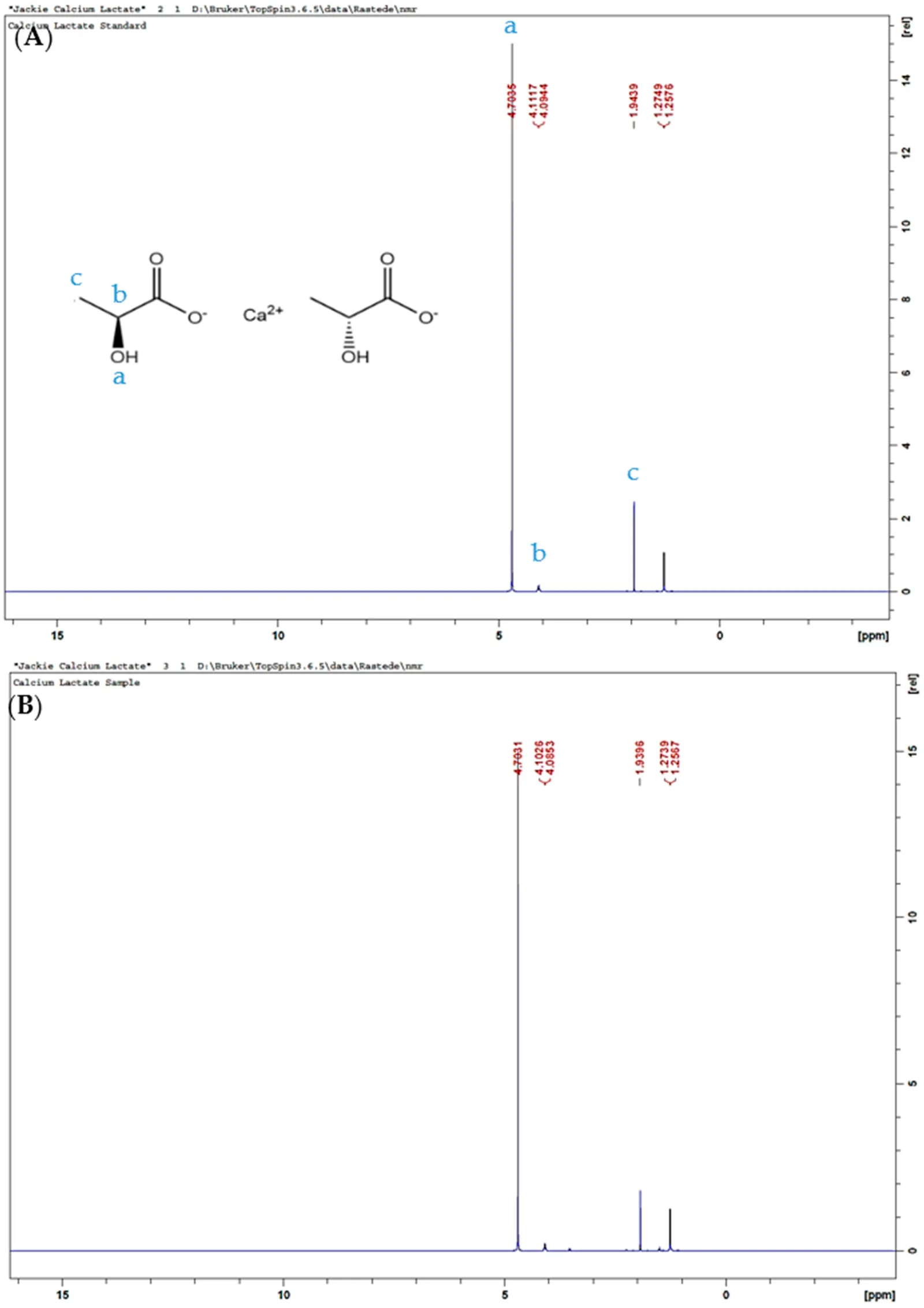
^1^H-NMR spectra of (**A**) Ca lactate standard and (**B**) recycled Ca lactate obtained after depolymerization of black PLA filaments in 50% ethanol.

For further confirmation of the identity and purity of the recycled Ca lactate, samples were characterized by ^1^H-NMR. As illustrated in Figure 6, the signals at 4.11 ppm (quartet, 1H) and 1.27 ppm (doublet, 3H), corresponding to the Ca lactate structure as reported in the literature, were the significant signals observed for the standard Ca lactate and recycled Ca lactate from black PLA filaments depolymerized in 50% ethanol. The singlet signal at 4.7 ppm corresponds to water. Nevertheless, the singlet signal observed at 1.94 ppm in the spectra of both the standard and recycled Ca lactate does not correlate with the structure of Ca lactate and could be a contaminant in the solvent used for analysis.

For acidity evaluation, all samples were neutralized with 0.05 ± 0.01 mL of NaOH, which indicated the absence of lactic acid or acidity. To investigate heavy metal content, 14 different heavy metals (Cr, Cd, Hg, Pb, Cu, Zn, As, Se, Rb, Sr, Sb, Ba, Bi and U) were analyzed. The samples’ total heavy metals content (1.7 ± 0.2 ppm) was below the USP requested limit of 20 ppm [14]. The hydration state of the recycled Ca lactate was determined by the percentage of mass loss upon drying. Comparing the results with the USP standards [14], it was indicated that the obtained Ca lactate was monohydrate in nature with an average mass loss (%) of 6 ± 3%. It is also worth noting that the slight shift observed in the position of the characteristic FTIR peaks in the recycled Ca lactate from that of the standard Ca lactate could be attributed to the difference in the hydration states of the recycled Ca lactate (monohydrate) and the standard one (pentahydrate).

### 3.4. Recovered Ca Lactate in the Recycled White Powder from PLA Depolymerization

A calibration curve was constructed using Ca^2+^ ion standards. Method validation was performed according to ICH Q2B guidelines for validation of analytical assays with respect to linearity, accuracy, precision (within and between days), limit of detection (LOD), and limit of quantification (LOQ). Table 3 shows a summary for validation results. The regression plot (Figure S4) showed good linearity with small intercept and good correlation coefficient (R^2^) of 0.9998.

Upon analysis of the recycled Ca lactate samples produced from our initial trials for studying the effect of aliphatic alcohol type and pigmentation color on the characteristics of produced Ca lactate, it was found that the pigmentation difference did not have a significant effect on the yield of Ca lactate where the yields’ standard deviation did not exceed 5%. The chain length of the aliphatic alcohol used also did not have a significant effect on the content of Ca in Ca lactate, showing a 64 ± 5%, 62 ± 4% and 61 ± 7% Ca^2+^ yield upon PLA depolymerization in 50% methanol, 50% ethanol and 50% isopropanol, respectively. Nevertheless, these values were low with respect to what was required by the USP or that contained in the commercially available Ca lactate that showed a Ca^2+^ yield of 99.8%. Thus, further modification was applied in the procedure for Ca lactate extraction while using 50% ethanol as the aliphatic alcohol since it had the upper hand in the yield% of PLA depolymerization. For instance, the acidified filtrate containing lactic acid and NaCl salt was completely dried to remove all water content, and lactic acid was separated and dissolved in 100 mL DI water followed by neutralization with CaCO_3_, filtration and concentration. The concentrated solution was then left overnight in the fridge to solidify and dried in the oven at 45 °C. Samples showed a significant increase in the average Ca ion recovery in recycled Ca lactate to 88 ± 3% instead of the 62 ± 4% indicating the success of the new extraction procedure and a room for continuous development to reach the 100%. The remaining 12% is assumed to be the NaCl salt that was not completely removed after the acidification of Na lactate with HCl. Further improvement in future work could include the increase in the filtration cycles to ensure the complete removal of the NaCl from lactic acid before proceeding with the following steps.

### 3.5. Quality Parameters of the Formulated Ca Lactate Tablets

#### 3.5.1. Tablet Size and Mass Uniformity

Six different formulations of Ca lactate tablets were manufactured to determine the ingredient ratios that will provide the best shape and size uniformity in addition to maximized Ca^2+^ content. At first, commercialized Ca lactate was used as the active ingredient for all formulations, and based upon its results, a few trials were selected to be manufactured using the recycled Ca lactate obtained from the depolymerization of black PLA filaments.

**Figure 7:**
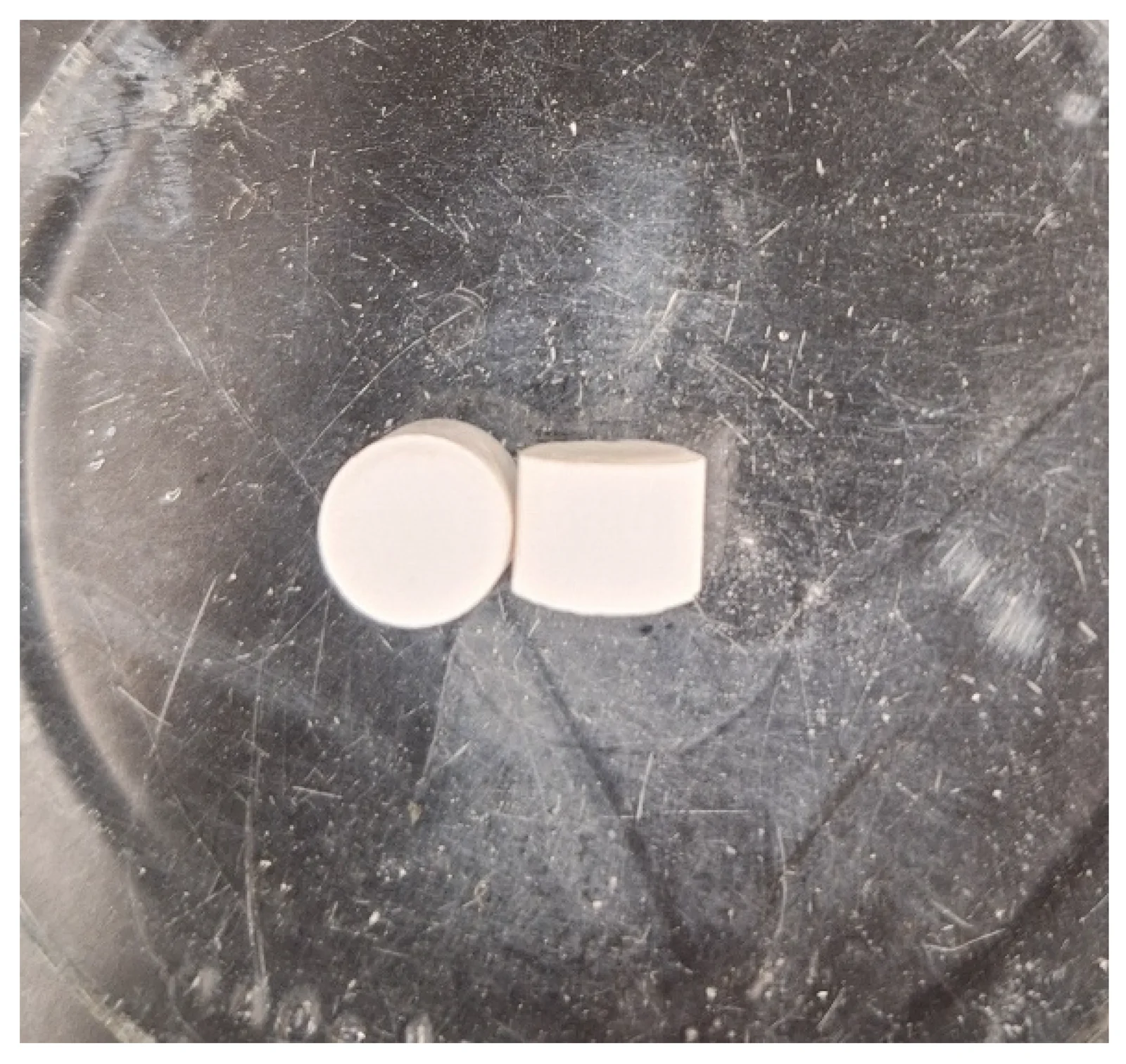
Image of manufactured tablets from recycled Ca lactate using the trial 6 formulation.

For all formulations (using commercial and recycled Ca lactate), round shape, white colored tablets with a flat surface, uniform appearance and intact edges were obtained. Figure 7 shows the tablets formulated using the trial 6 formulation.

The average sizes of the tablets (thickness 6 mm and diameter 7 mm) are evidence of the uniformity in the structure of the material for direct compression and a well-conducted process.

Table 4 illustrates the mass of the formulated tablets from both commercial Ca lactate and recycled Ca lactate. The mass variation for all formulations was small where no tablet’s mass was outside the limits of 85–115% of the average mass [15]. This indicated the consistency of the dosage obtained. Nevertheless, it was noticed that the average mass of the recycled Ca lactate was slightly higher than that of commercial Ca lactate for the same trial, which could be attributed to the difference in the molecular weights of both powders or their flowability inside the press.

#### 3.5.2. Tablet Calcium Ion Content

The manufactured tablets were evaluated for Ca^2+^ ion content using the developed AAS assay. As shown in Table 4, the results for all formulations comply with USP criteria by having low SD values, which demonstrates the homogeneity between the tablets of the same formulation. For both the commercial Ca lactate tablets and recycled Ca lactate tablets, the highest Ca loading was found in trial 6 with 28.2 ± 0.8 mg Ca/tablet and 29.3 ± 0.2 mg Ca/tablet, respectively, while the lowest loading was found for trial 1. Such a difference could be attributed to the initial Ca lactate content that was mixed to manufacture the tablet; for example, trial 6 Ca lactate content was 1.5 times that of trial 1. Accordingly, the trial 6 formulation was selected for further investigation concerning the dissolution kinetics and toxicity evaluation.

Typically, ingestion of six small tablets (340 mg each) of recycled Ca lactate supplements would be equivalent to 18% of the daily required dietary value of Ca (DV%). The same DV% is provided by two 650 mg commercially available Ca lactate tablets [17].

Such results indicate that the recycled Ca lactate supplements can provide a product with good potentiality, given that the source of Ca lactate is waste and that the price of production of recycled 0.76–0.93 kg Ca lactate is around 3.02$ as elaborated in our previous study, compared to $2–5/kg for the production cost of commercially available Ca lactate [3]. This is in addition to the environmental sustainability gains from this combined recycling/upcycling approach.

### 3.6. In Vitro Dissolution Kinetics of Recycled Ca Lactate Tablets

**Figure 8:**
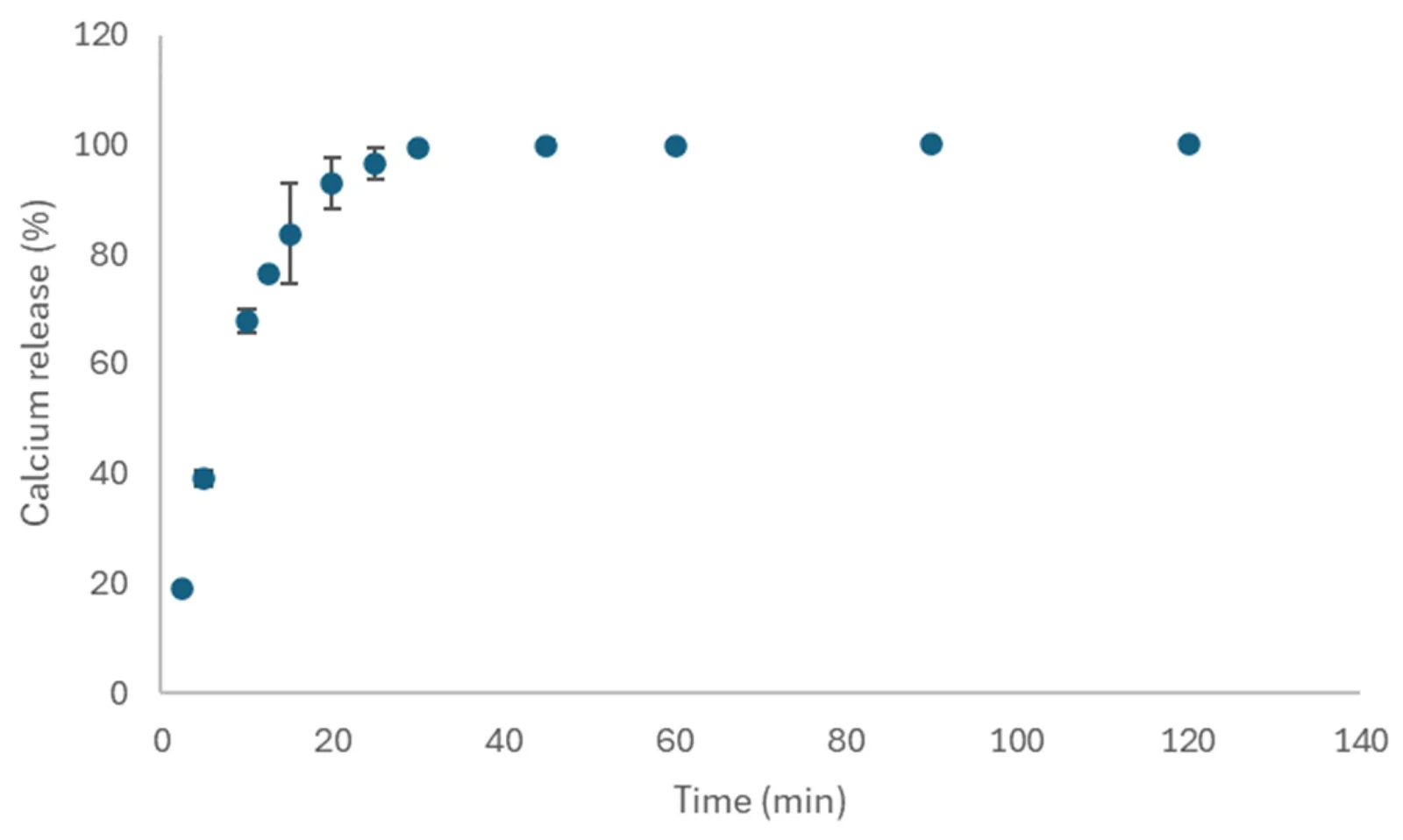
Dissolution profile of recycled Ca lactate tablets.

The in vitro dissolution testing (Figure 8) demonstrated a fast and complete release of Ca^2+^ from the recycled Ca lactate tablets, with 100% release achieved within 40 min.

The Ca^2+^ release data were fitted to a range of kinetic models: zero-order, first-order, Weibull, Higuchi, Hixson–Crowell, and Korsmeyer–Peppas models through KinetDS software. Version 3.0 For each model, correlation coefficients (R^2^) and root mean square error (RMSE) were determined (see Table 5). The selected release order followed the model that provided R^2^ values closer to unity and the smallest RMSE.

Based on the obtained results in Table 5 for R^2^ and RMSE, it was decided that the Weibull function can be efficiently used to reproduce the release process. The Weibull model formula is expressed in Equation (6) [18] and the Weibull best fit for the Ca^2+^ release is shown in Figure S5.

Percentage of Ca release (%) = 100 × (1 − exp^−((t−lag)β)/K^) (6)

where lag time = 0, and β and K are constants related to the specific release mechanisms.

A value of β < 0.75 is associated with Fickian diffusion, while a combined mechanism (Fickian diffusion and swelling-controlled transport) is associated with β values in the range 0.75 < β < 1. For the shape factor, β = 1, the release profile follows an exponential curve (similar to first-order kinetics), while when β > 1, the release mechanism is quite complex: the rate of release initially increases non-linearly up to the inflection point and thereafter decreases asymptotically [19,20].

For our recycled Ca lactate tablets, the shape parameter β, had a value of 1.005, which corresponds to an exponential or near-first-order dissolution curve that starts with a steep, rapid release that gradually flattens out as the remaining concentration of Ca lactate in the tablet decreases over time. [20]. The characteristic K, which represents the time required to release 63.2% of the total Ca^2+^ load, was estimated to be 14.7 min, indicating a rapid overall release rate. Such a finding was also confirmed by the calculation of the model-independent parameters based on the 12 time points; Dissolution Efficiency (DE) and Mean Dissolution Time (MDT) were represented by Equations (S4) and (S5), respectively [18]. After the 40 min release experiments, the dissolution efficiency was estimated to be 81.0% with an MDT of 8.56 min.

### 3.7. Cytotoxicity Evaluation of Recycled Ca Lactate Tablets

The cytotoxic potential of the calcium lactate tablet extract was evaluated in THLE-2 human hepatocytes following 48 h of exposure. Cells were treated with 0.0165, 0.033, or 0.066 mg/mL extract, corresponding to the low-, mid-, and high-dose groups, respectively. The tested concentrations were selected to encompass tablet-equivalent exposures derived from the recommended human intake and corresponding to tablet-equivalent exposure in an average human.

**Figure 9:**
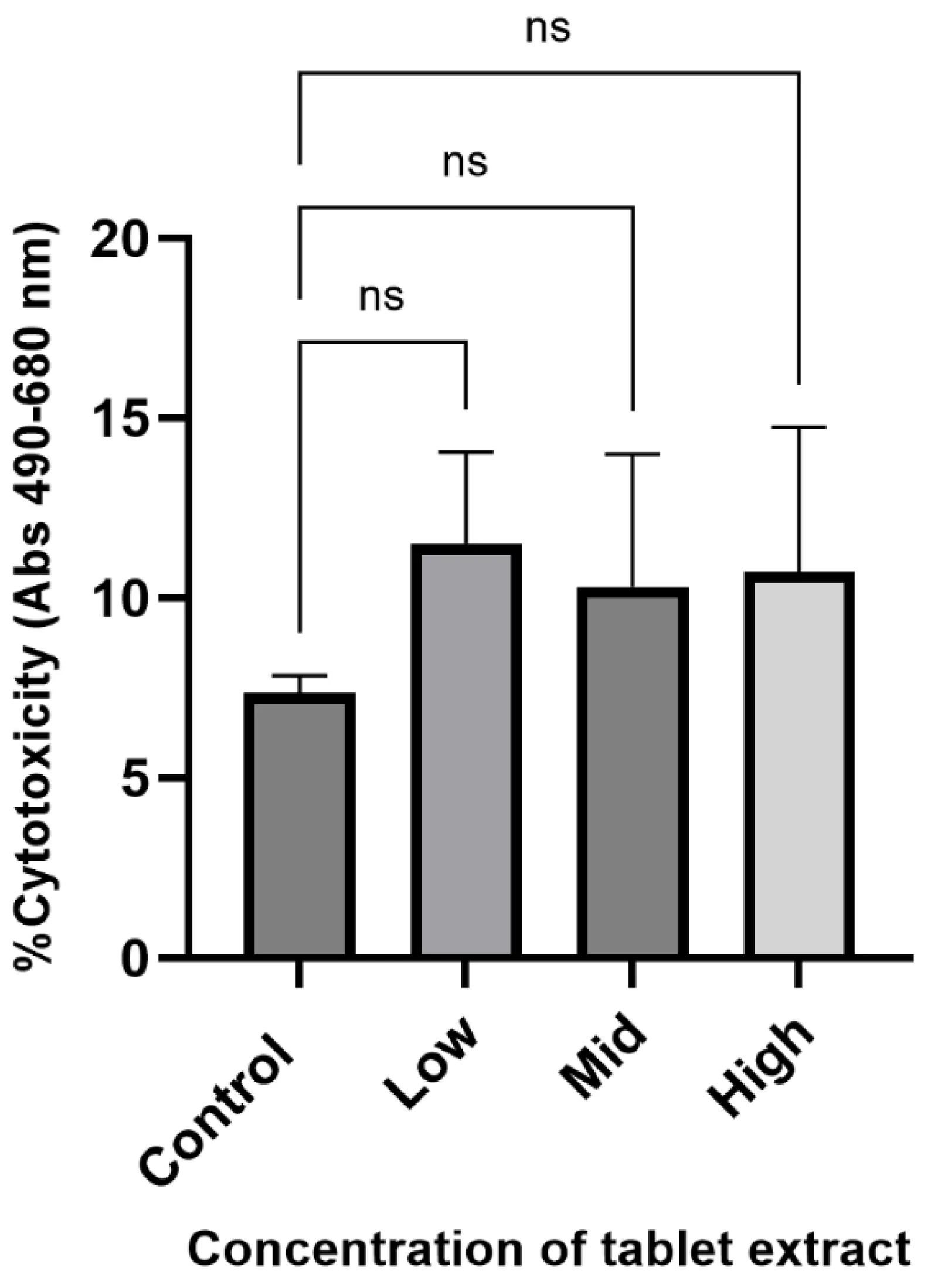
Effect of calcium lactate tablet extract on cytotoxicity in THLE-2 cells.

As shown in Figure 9, exposure to calcium lactate tablet extract did not significantly alter cytotoxicity, as determined by LDH activity, across the tested concentrations (ordinary one-way ANOVA, *F* (3, 8) = 1.080, *p* = 0.4111) [21,22]. Although a modest numerical increase in cytotoxicity was observed in the low-dose group relative to the untreated control, Dunnett’s multiple-comparisons test indicated that none of the treatment groups differed significantly from the untreated control (*p* > 0.05). Additionally, no concentration-dependent increase in cytotoxicity was observed. These findings indicate that exposure to 0.0165–0.066 mg/mL aqueous calcium lactate tablet extract for 48 h did not induce significant cytotoxicity in THLE-2 cells under the conditions tested.

These findings suggest that the aqueous calcium lactate tablet extract was not overtly cytotoxic to THLE-2 cells at concentrations ranging from 0.0165 to 0.066 mg/mL under the conditions examined. The absence of a concentration-dependent increase is particularly notable, as a treatment-related cytotoxic effect would generally be expected to become more pronounced with increasing exposure. The modest elevation observed at the lowest concentration therefore likely reflects experimental variability rather than a biologically meaningful treatment effect, particularly because this response was not reproduced at the intermediate or highest concentrations.

Collectively, these results support the cytocompatibility of the calcium lactate tablet extract within the concentration range evaluated. However, the findings should be interpreted within the limits of the experimental model. LDH release primarily reflects loss of plasma membrane integrity and therefore provides an assessment of overt cytotoxicity rather than more subtle alterations in cellular metabolism, oxidative stress, proliferation, or signaling [22]. In addition, the present analysis was limited to a 48 h exposure period. Further evaluation using complementary cellular endpoints and additional exposure durations would therefore provide a more comprehensive assessment of the biological response to the tablet-derived extract.

THLE-2 cells were left untreated (control) or treated with 0.0165 (low), 0.033 (mid), or 0.066 mg/mL (high) aqueous calcium lactate tablet extract for 48 h. Cytotoxicity was assessed by LDH release using the CyQUANT LDH Cytotoxicity Assay. Data are presented as the mean ± SD from three independent experiments, with three technical replicates performed for each condition. Statistical significance was assessed using ordinary one-way ANOVA followed by Dunnett’s multiple-comparisons test comparing each treatment group with the untreated control. No significant differences were detected among groups (*F*(3, 8) = 1.080, *p* = 0.4111); ns—not significant.

## 4. Conclusions

This study provided additional evidence that our proposed alkaline hydrolysis in alcoholic media is an efficient method for PLA chemical recycling, as it can be performed under mild conditions at atmospheric pressure. It was also found that, irrespective of pigmentation color, the highest PLA conversion (94%) and monomer yield (20.6 mmol) were obtained in 50% ethanol media. A small PLA filament size (0.5 cm) had a higher (1.5×) reaction rate constant than that of longer filaments, indicating that filament size affected the rate of PLA depolymerization using the proposed method.

Atomic absorption spectroscopy indicated that 88% of elemental calcium was recovered in the obtained Ca lactate powder. The FTIR and NMR confirmed the identity and purity of calcium lactate. The acidity, heavy metal content and hydration state of the recycled calcium lactate proved its efficiency for use in the tablet formulation according to US Pharmacopeia standards.

The quality of the formulated calcium lactate tablets with respect to calcium loading, release kinetics and cytotoxicity was assessed. The results indicated that high-quality calcium lactate tablets were manufactured with uniform shape, dimensions and calcium content reaching 29 mg Ca/tablet. The in vitro dissolution testing showed an immediate release behavior in less than 40 min and a dissolution efficiency of 81%, within the limits imposed by the US Pharmacopeia. The cytotoxicity test performed for the Ca lactate tablets on human hepatocytes indicated their cytocompatibility within the evaluated concentrations.

In conclusion, the recycled calcium lactate produced from PLA depolymerization serves as a good candidate for the pharmaceutical industry as it is environmentally friendly, cost-effective, has acceptable properties according to the US Pharmacopeia, and can form good quality drug delivery systems.

## Figures and Tables

**Table 1:** Ca lactate tablet formulation trials represented as ratios.

Sample	Calcium Lactate	Microcrystalline Cellulose 302	Sodium Starch Glycolate	Magnesium Stearate	Total
**Trial 1**	0.62	0.36	0.01	0.01	1.00
**Trial 2**	0.66	0.35	0.01	0.01	1.00
**Trial 3**	0.69	0.29	0.01	0.01	1.00
**Trial 4**	0.74	0.24	0.01	0.01	1.00
**Trial 5**	0.79	0.19	0.01	0.01	1.00
**Trial 6**	0.93	0.05	0.01	0.01	1.00

**Table 2:** Rate constants (k) of white PLA depolymerization in 50% ethanol at different filament size.

Filament Size (cm)	k (min^−1^)	Correlation Coefficient (R^2^)
0.5	0.0671	0.9731
1.0	0.0578	0.9866
2.0	0.0474	0.9427

**Table 3:** Atomic Absorption Spectroscopy method validation for quantitation of Ca^2+^ in Ca lactate.

Item	Ca^2+^ Ions
**Wavelength of absorption (nm)**	422.7
**Range of linearity**	4–14 ppm
**Regression equation**	Abs = 0.0682 C_ppm_ + 0.035
**Regression coefficient (R^2^)**	0.9998
**LOD (ppm)**	0.3
**LOQ (ppm)**	1
**Sb (SD of slope)**	0.0004
**Sa (SD of intercept)**	0.004
**Accuracy mean ± SD (%)**	100 ± 1
**Repeatability (%RSD, *n* = 6)**	0.3
**Precision**	
**Intraday (%RSD, *n* = 3)**	0.4–0.6
**Interday (%RSD, *n* = 3)**	0.02–0.5

**Table 4:** Mass and Ca content of commercial Ca lactate and recycled Ca lactate tablets.

Sample	Commercial Ca Lactate Tablet Mass (mg ± S.D, *n* = 5)	Recycled Ca Lactate Tablet Mass (mg ± S.D, *n* = 5)	Commercial Ca Lactate Tablet Ca^2+^ Content (mg ± S.D, *n* = 3)	Recycled Ca Lactate Tablet Ca^2+^ Content (mg ± S.D, *n* = 3)
**Trial 1**	300 ± 10	320 ± 10	22.0 ± 0.8	21.8 ± 0.4
**Trial 2**	300 ± 10	Not tested	24 ± 3	Not tested
**Trial 3**	310 ± 6	Not tested	24.1 ± 0.3	Not tested
**Trial 4**	309 ± 3	Not tested	25.7 ± 0.1	Not tested
**Trial 5**	310 ± 10	330 ± 20	26.4 ± 0.3	26.7 ± 0.4
**Trial 6**	310 ± 7	320 ± 8	28.2 ± 0.8	29.3 ± 0.2

**Table 5:** A list of release models and their determination coefficients of linearization for Ca^2+^ release from the recycled Ca lactate tablets.

Release Model	R^2^	RMSE
**0 order**	0.345	20.8
**1st order**	0.260	24.1
**Weibull**	0.954	2.00
**Higuchi**	0.432	30.8
**Hixson–Crowell**	0.289	22.3
**Korsmeyer–Peppas**	0.717	20.0

## Data Availability

All data generated or analyzed during this study are included in the article (and its Supplementary Information Files).

## Supplementary Materials

The following supporting information can be downloaded at: https://www.mdpi.com/article/10.3390/polym18192422/s1, S1: Instrumentation; S2: First order reaction kinetics equation for PLA filaments depolymerization; Figure S1: FTIR spectra of [a] Ca lactate standard and [b] Recycled Ca lactate obtained after depolymerization of PLA filaments in 50% methanol; Figure S2: FTIR spectra of [a] Ca lactate standard and [b] Recycled Ca lactate obtained after depolymerization of PLA filaments in 50% ethanol; Figure S3: FTIR spectra of [a] Ca lactate standard and [b] Recycled Ca lactate obtained after depolymerization of PLA filaments in 50% isopropanol; Figure S4: Calibration curve for Calcium ion quantitation using Atomic absorption method; Figure S5: Weibull model fitting for Ca release from recycled Ca lactate tablets; Equation S1: for Dissolution Efficiency (DE); Equation S2: for Mean Dissolution Time (MDT).
