# Supplementary material for: Preparation and Characterization of Immediate-Release Tablets Containing Calcium Lactate Recovered from Chemically Recycled Poly(lactic acid) 3D Printing Filaments
Source: Polymers (Basel). 2026 Oct 4;18(19):2422. doi: 10.3390/polym18192422 (PMC13666945; doi:10.3390/polym18192422)
Supplement: Supplementary file 1 [file polymers-18-02422-s001.zip › polymers-4605625-supplementary.pdf]

Supplementary information

Preparation and Characterization of Immediate-Release Tablets Containing Calcium Lactate Recovered from Chemically Recycled Poly(lactic acid) 3D Printing Filaments

Olivia A. Attallah 1,*, Dario Brandao 2, Jacqueline Tran 1, Irvin Rivera 1, Cindy A. Thomas-Charles 3, Michael R. O'Krepky 4 and Mathew Mannon 2

[1] *Chemistry Department, University of Hartford, West Hartford, CT 06117, United States*

[2] *Geoscience Department, Union College, Schenectady, NY 12308, United States*

[3] *Biology Department, University of Hartford, West Hartford, CT 06117, United States*

[4] *Chemistry Department, Union College, Schenectady, NY 12308, United States*

*Correspondence: soliman@hartford.edu; soliman@hartford.edu or olivia.adly@gmail.com

Supplementary information Page

S1 Instrumentation 3

S2 First order reaction kinetics equation for PLA filaments depolymerization 4

Figure S1: FTIR spectra of [a] Ca lactate standard and [b] Recycled Ca lactate obtained after depolymerization of PLA filaments in 50% methanol 5

Figure S2: FTIR spectra of [a] Ca lactate standard and [b] Recycled Ca lactate obtained after depolymerization of PLA filaments in 50% ethanol 6

Figure S3: FTIR spectra of [a] Ca lactate standard and [b] Recycled Ca lactate obtained after depolymerization of PLA filaments in 50% isopropanol 7

Figure S4: Calibration curve for Calcium ion quantitation using Atomic absorption method 8

Figure S5: Weibull model fitting for Ca release from recycled Ca lactate tablets 9

Equation S1: for Dissolution Efficiency (DE) 10

Equation S2: for Mean Dissolution Time (MDT) 10

References 11

S1 Instrumentation

The chemical structure of the lactic acid in the form of Ca lactate obtained from recycling of PLA was confirmed by recording their IR and ^{1}H-NMR spectra. For functional groups analysis, an FT-IR spectrophotometer with Diamond Anvil ATR from Thermo-electron, USA was used. The technique used was that of Attenuated Total Reflectance (ATR-FTIR) since solid materials were used. The resolution of the instrument was 4 cm^{-1}. The recorded wavenumber range was from 500 to 4000 cm^{-1} and 16 spectra were averaged to reduce the noise. To confirm the chemical structure identity, ^{1}H-NMR analysis was performed on Bruker Avance III, USA using the operating frequency of 400 MHz at 25°C in D_2O.

S2 First order reaction kinetics equation for PLA filaments depolymerization [1]

PLA filaments depolymerization is controlled by first order reaction kinetics with respect to PLA amount in 50% ethanol as shown in equation (A)

$$\frac{d(C_{PLA\,filaments})}{dt} = -kC_{PLA\,filaments} \qquad \text{(A)}$$

where k indicates the constant rate of reaction and $C_{PLA\,filaments}$ indicates the mass of PLA filaments in 50%ethanol at time t.

The concentration of PLA filaments can then be calculated as via equation (B),

$$C_{PLA\,filaments} = C_{PLA\,filaments\,0}\,(1 - X) \qquad \text{(B)}$$

Where X represents the conversion of PLA filaments. The integrated linearized form of that equation is shown in equation (C).

$$\ln \frac{1}{(1-x)} = kt \qquad \text{(C)}$$

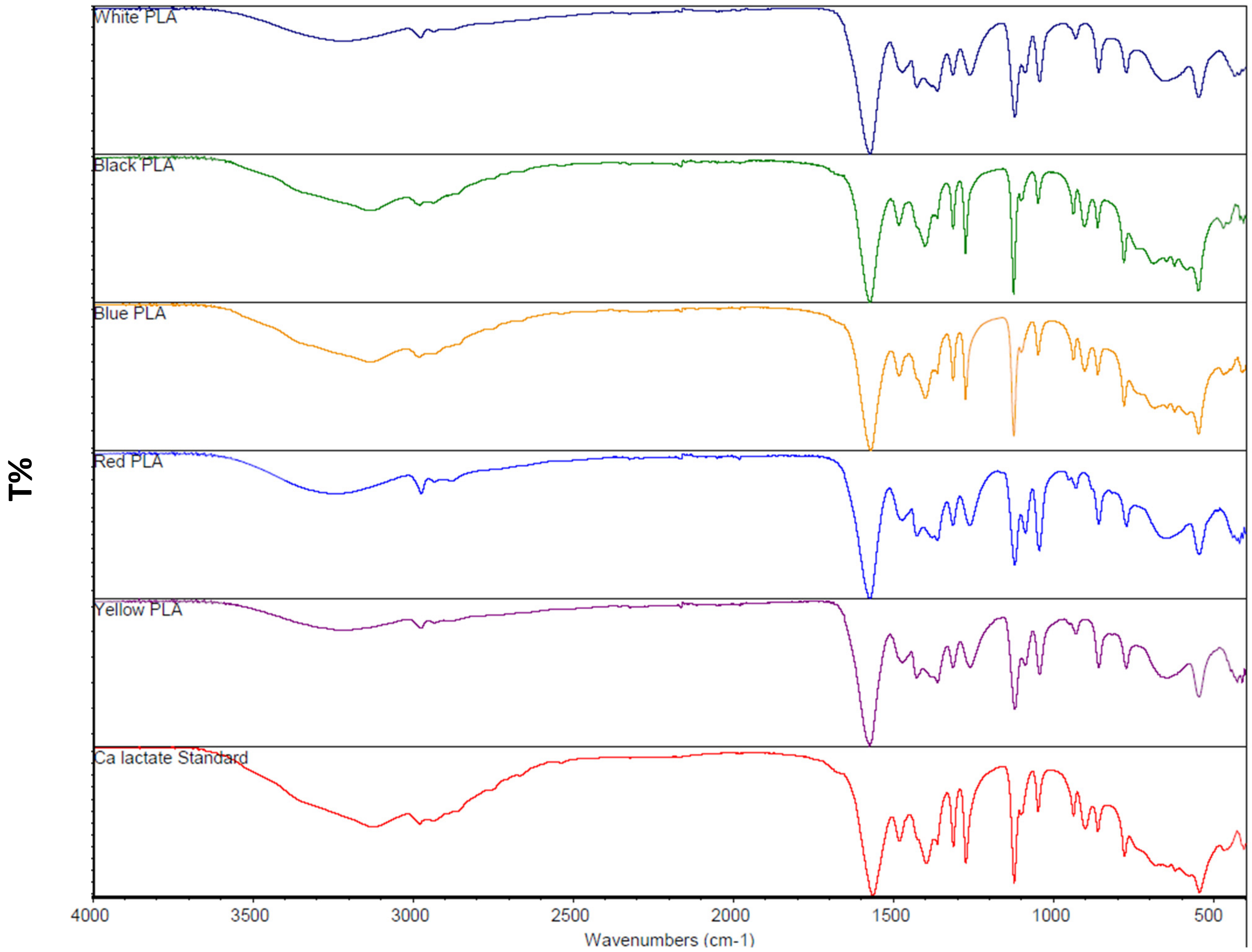

Figure S1: FTIR spectra of [a] Ca lactate standard and [b] Recycled Ca lactate obtained after depolymerization of PLA filaments in 50% methanol

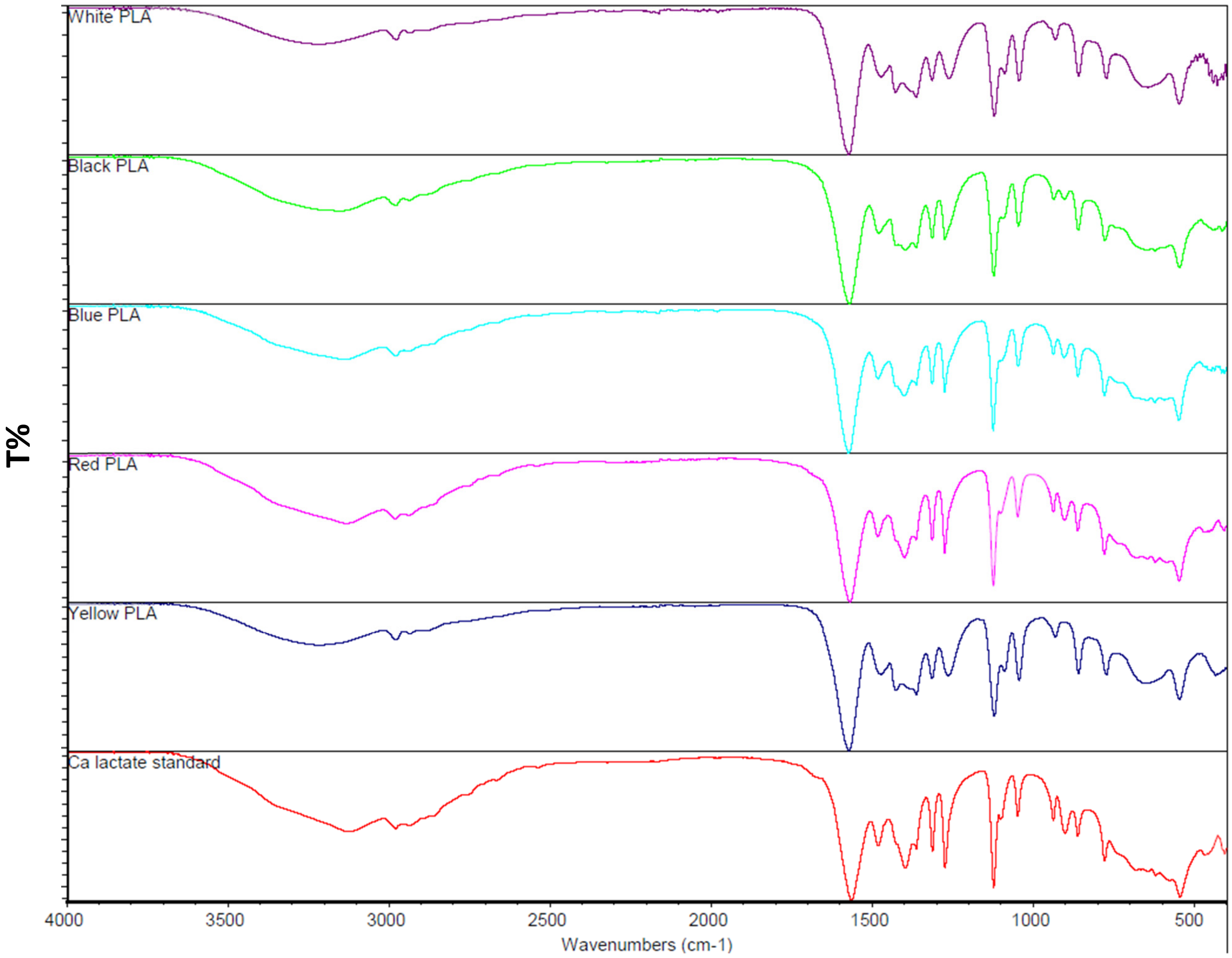

Figure S2: FTIR spectra of [a] Ca lactate standard and [b] Recycled Ca lactate obtained after depolymerization of PLA filaments in 50% ethanol

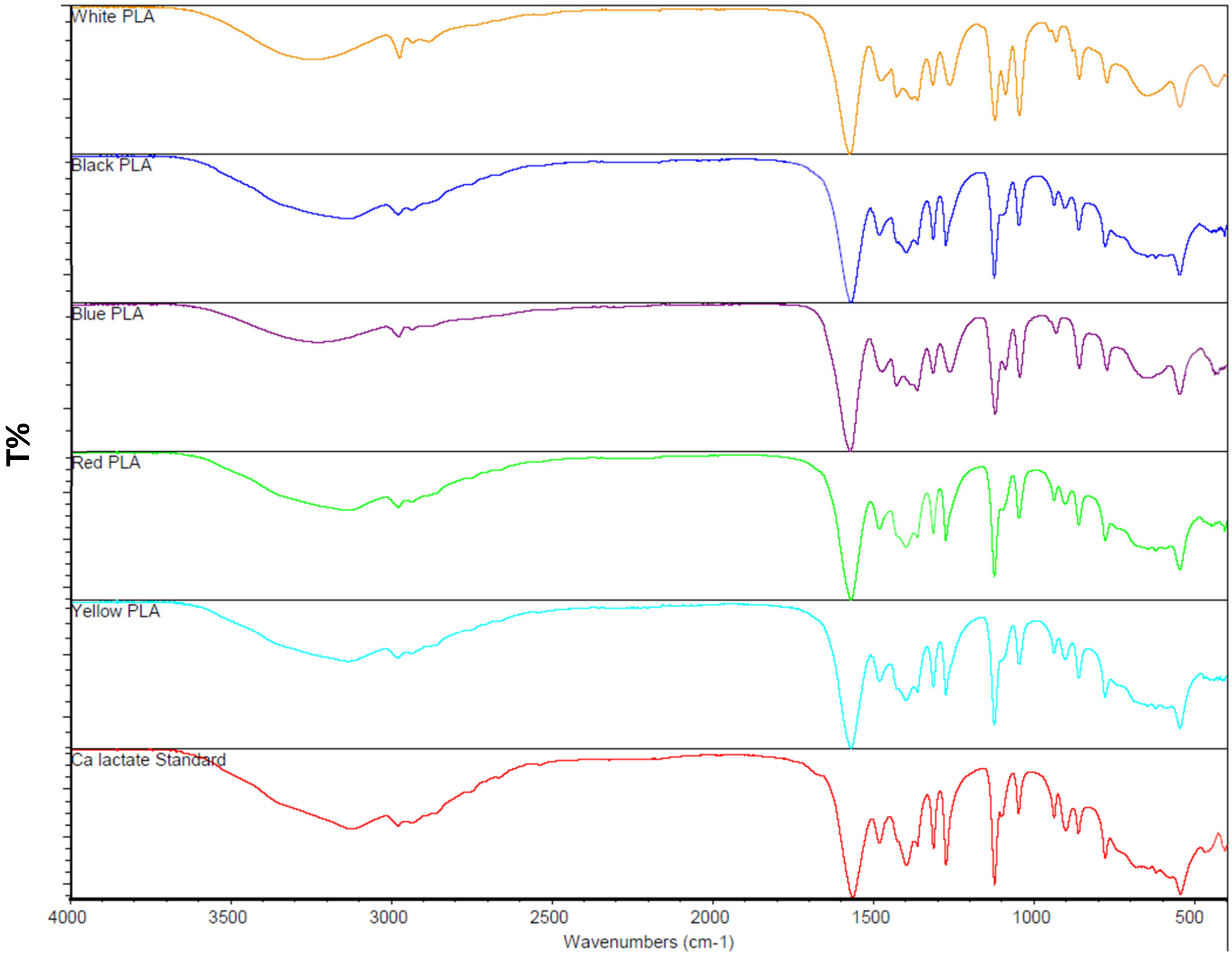

Figure S3: FTIR spectra of [a] Ca lactate standard and [b] Recycled Ca lactate obtained after depolymerization of PLA filaments in 50% isopropanol

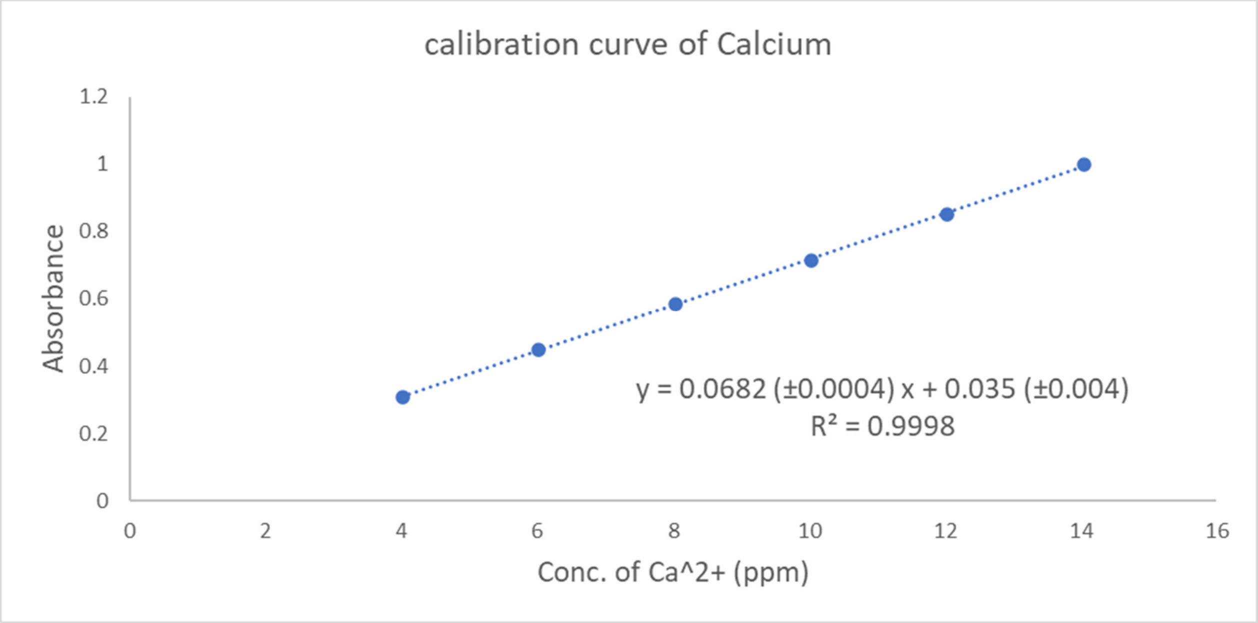

Figure S4: Calibration curve for Calcium ion quantitation using Atomic absorption method

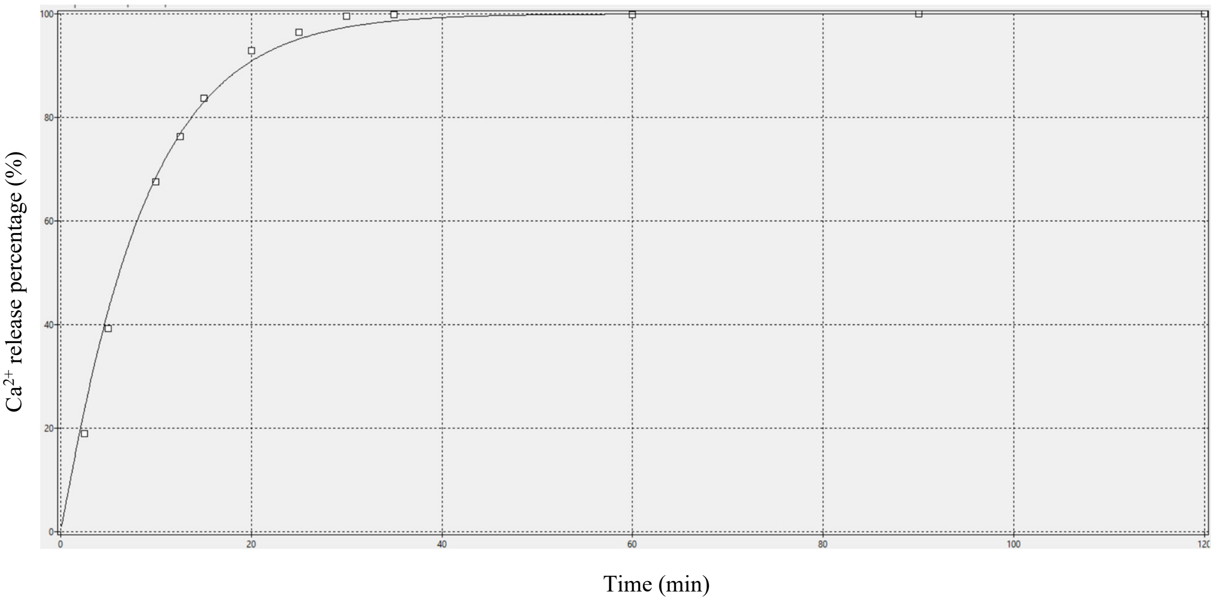

Figure S5: Weibull model fitting for Ca release from recycled Ca lactate tablets

Equation S1: for Dissolution Efficiency (DE)[2]

$$DE = \frac{\int_0^t Q\,dt}{Q_{100} \times t} \times 100$$

where:

Q100 is maximum amount of drug released (= 100%)

Equation S2: for Mean Dissolution Time (MDT)[2]

$$MDT = \frac{\sum_{j=1}^{n} t_j^{AV} \times \Delta Q_j}{\sum_{j=1}^{n} \Delta Q_j}$$

where:

$\Delta Q = Q_{(t)} - Q_{(t-1)}$

$t_j^{AV} = (t_i + t_{i-1})/2$

n – amount of timepoints

References

[1] O.A. Attallah, V. Palkova, R. Vij, Single Step Depolymerization of Multiple Polyesters in Poly(Lactic Acid) Mixed Plastics: Process Optimization, Pure Monomers Extraction and Kinetics Evaluation, J. Polym. Environ. 2025 334 33 (2025) 1897–1915. https://doi.org/10.1007/S10924-025-03523-6.

[2] Aleksander Mendyk, Renata Jachowicz. Unified methodology of neural analysis in decision support systems built for pharmaceutical technology. Expert Systems with Applications 32 (2007) 1124–1131.
